# Benchmarking state of the art website embedding methods for effective processing and analysis in the public sector

**DOI:** 10.1007/s10844-025-00954-4

**Published:** 2025-06-13

**Authors:** Jonathan Gerber, Jasmin Saxer, Bruno Kreiner, Andreas Weiler

**Affiliations:** Institute of Computer Science, Zurich University of Applied Science, ZHAW, Obere Kirchgasse 2, Winterthur, 8400 Zurich Switzerland

**Keywords:** Embedding evaluation, Website embedding, Website classification, Content monitoring, Cluster evaluation

## Abstract

The ability to understand and process websites is crucial across various domains. It lays the foundation for machine understanding of websites. Specifically, website embedding proves invaluable when monitoring local government websites within the context of digital transformation. In this paper, we present a comparison of different state-of-the-art website embedding methods and their capability of creating a reasonable website embedding for our specific task. The models consist of visual, mixed, and textual-based embedding methods. We compare the models with a baseline model which embeds the header section of a website. We measure the performance of the models using zero-shot and transfer learning. We evaluate the performance of the models on three different datasets. Additionally to the embedding scoring, we evaluate the classification performance on these datasets. From the zero-shot models Homepage2Vec with visual, a combination of visual and textual embedding, performs best in general over all datasets. When applying transfer learning, TF-IDF & FNN, a text based model, outperforms the others in both cluster scoring as well as precision and F1-score in the classification task. However, time is an important factor when it comes to processing large data quantities. Thus, when additionally considering the time needed, our baseline model is a good alternative, being 1.88 times faster with a maximum decrease of 10 % in the F1-score.

## Introduction and motivation

From individuals seeking information to machine learning marvels like chatbots and trading algorithms, countless entities rely on the data ocean, known as the World Wide Web. In this landscape, website monitoring plays a crucial role. By analyzing constantly changing internet data, these models handle diverse tasks ranging from event detection and price tracking to ensuring compliance with evolving policies. One noteworthy example is the European Union’s 2016 Directive on website accessibility for public services (Directive (EU) 2016/2102). Monitoring tools can help to ensure these regulations are upheld, promoting an inclusive digital space for all.

When looking at the significance of website monitoring, it becomes clear that tools are vital for understanding the broader landscape of digital transformation. We seek to analyze websites from local governments across Europe with the end goal of assessing their digitalization. While the assessment itself is not part of this paper, we set the foundation for an ongoing interdisciplinary research project between computer and political science called Digilog^1^. We provide more detailed information about the project in Gerber et al. (2024) as well as in Gerber et al. (2024b). This work is supported by Grant No. GR 200839 of the Swiss National Science Foundation (SNF) and German Research Foundation (DFG) for the research project “Digital Transformation at the Local Tier of Government in Europe: Dynamics and Effects from a Cross-Countries and Over-Time Comparative Perspective (DIGILOG)”.

There is already work claiming to measure the level of digital transformation within local governments. García-Sánchez et al. (2013) present an analysis of the development of e-governments of 102 Spanish municipalities and Pina et al. (2007) conduct an empirical study about the effect of e-government on transparency, openness and hence accountability in 15 countries of the EU and a total of 318 government web sites. There are countless other approaches to assess the digital maturity of local governments based on the provided information on their websites (e.g., Andersen and Henriksen (2006); Layne and Lee (2001); Windley (2002)). Patergiannaki and Pollalis provide a recent overview on the different evaluation models introduced in this field so far (Patergiannaki and Pollalis, 2023). However, almost all of the work is based on manual inspection of a website, which limits the capability of such an assessment dramatically in terms of the number of assessments. Thus our contribution aims to lay a foundation to the effective and efficient assessments of websites in the field of public sectors.

When analyzing websites over time, mutations such as domain changes or emerging of new websites frequently occur. To maintain an up-to-date list of municipality URLs, we propose a method to verify websites’ authenticity, particularly distinguishing between governmental and tourism sites. Our research reveals that crawlers often mistake tourism sites for government ones. This classification task as well as all other downstream tasks (e.g. e-service detection, analysis of digital transformation, etc.) require a numerical representation of the website. However, an accurate representation of websites using numerical embeddings derived from Natural Language Processing and Computer Vision models is challenging. We evaluate the performance of pre-trained model embeddings in three different tasks:

- Binary classification of websites divided into municipality and non-municipality classes, which we will call Municipality classification task (MCT).
- Binary classification, which consists of the detection of e-forms and e-services on municipality websites, which we call eService classification task (SCT).
- Multiclass classification of websites within the public sector, which we will call Public sector classification task (PCT).

This work is a successor of Gerber et al. (2024a). We supplemented the MCT dataset in this work discussed and added two more datasets as well as classification tasks. We provide a detailed analysis of the results and a comprehensive comparison of the different performances. We also provide our code with documentation^2^

## Related work

Websites use both visual (images, rendered HTML code) as well as textual (floating text) features to present content to users. To extract the full depth of information, an embedding model should be capable of processing both visual and textual data. Thus it is not surprising that Large Language Models (LLM) and Convolutional neural networks (CNN) are often used in recent work. There are also other classical machine learning approaches that rely more on feature engineering. However, they do not generalize as well as the state-of-the-art models, due to their lack of flexibility when it comes to structural changes of an HTML page. There exists a large amount of related work in the field of text-based-only embedding and classification of websites. Hashemi (2020), Kowsari et al. (2019) and Minaee et al. (2021) provide surveys on past work and discuss different approaches on website embedding. We only mention a selection of the recent work which is related to the approaches applied in this work. Visual-only based classifications are in many cases applied to the detection of harmful content. Whether detection of propaganda of terrorism (Hashemi and Hall, 2019), alcohol, adult content, weapons (Akusok et al., 2015; Espinosa-Leal et al., 2021) or just food, fashion and landscapes (López-Sánchez et al., 2017), the classes all have distinctive visual features. However, in many cases, these approaches cannot distinguish visually similar pages (e.g. municipality homepage vs. tourism page about the same municipality).

In the field of text-based website embedding/classification, there are approaches that rely on classical machine learning (Bhalla and Kumar, 2016; Matošević et al., 2021). However, the majority is based on neural networks or transformers. There are several RNN and LSTM-based approaches (Buber and Diri, 2019; Lin et al., 2020; Nandanwar and Choudhary, 2021; Zhou et al., 2021) to embed websites. Lin et al. (2020) and Zhou et al. (2021) additionally combine their BiLSTM approach with a CNN. There are different transformer based approaches (Chen et al., 2021; Gupta and Bhatia, 2021; Li et al., 2022; Nandanwar and Choudhary, 2023). We summarize the two most relevant approaches for our topic for each group (textual-/visual-based). Li et al. (2022) propose MarkupLM, a pre-trained LLM for document understanding tasks based on the actual text as well as the markup language. The model is based on the BERT architecture. They add the additional XPath embedding to the embedding layer, which is based on various features to identify the target leaf. The pre-training objectives of the models are Masked Markup Language Modeling (prediction of text token of DOM tree leaf), Node Relation Prediction (e.g., child, sibling, etc.), and Title-Page Matching. They compare their two models (base and large) with previous models such as FreeDOM-Full (Lin et al., 2020), SimpDOM (Zhou et al., 2021), and others on the SWDE dataset considering the F1-score. They also compare their models with BERT base, RoBERTa base, and ELECTRA large models from Chen et al. (2021) on the WebSRC dataset. Their large model outperforms every other model in every aspect, while their base model outperforms the others in most cases. The proposed models are available only in English. Nandanwar and Choudhary (2021) propose a classification model based on GloVe and a BiLSTM for categorizing. They test the model on the WebKB data set as well as the DMOZ dataset. They further compare their model against the ensemble model of Gupta and Bhatia (2021) and a Support Vector Machine web page classification approach (Bhalla and Kumar, 2016). In most cases, the proposed model outperforms the other models.

There also exists research on mixed approaches. Bruni and Bianchi (2020) introduce a procedure for website classification that leverages both textual and visual features. They compare different classification algorithms to identify e-commerce services on web pages. The classification approach they propose is highly sophisticated and may not align with our specific needs as they assume classes to have certain attributes such as those related to e-commerce services. Lugeon et al. (2022) propose a language-agnostic website embedding for classification tasks. With their introduced homepage embedder “Homepage2vec” they create a multilingual embedding based on word embeddings of the textual content (the first 100 sentences), the metadata tags (title, description, keywords, etc.), and also the visual appearance (screenshot) and other features such as domain name. The numerical features are concatenated and processed by a neural network. They are then used for classifying the website into 14 different classes (art, business, computers, games, etc.). While the feature embeddings seem to effectively capture the essence of the homepage, the model is constrained by a narrow range of broad classes.

## Methods

In this section, we clarify which pre-trained models we used for embedding, how we applied transfer learning, and how we evaluated the models’ embeddings.

As mentioned in Section 2, there are mainly three different approaches to embed websites: textual, visual, and combined methods. We apply two recently published methods and evaluate their performance on our datasets, which are described in Section 3.2. We selected Homepage2vec (Lugeon et al., 2022) and MarkupLM (Li et al., 2022) due to their performance and reproducibility. Both approaches leverage the deeper semantic understanding embedded within Markup Documents. MarkupLM incorporates the embedding of XPath and tags as features, while Homepage2vec integrates visual features alongside specific data from a Markup document, including keywords and descriptions found in the meta tag section. Notably, both authors provide a library or GitHub repository for applying their models. To accommodate the absence of a multilingual version for the MarkupLM model, text components were translated into English before being used for embedding.

*Homepage2vec* We used the Homepage2vec (Lugeon et al., 2022) library^3^ and its ready-made feature extractors. We slightly changed the way Homepage2vec retrieves websites. Namely, we allow for redirects using requests. If requests cannot fetch a site, we use Selenium with a headless Chrome web driver. Homepage2vec offers two options: Either use the visual embeddings using screenshots of the websites or simply leave them out. Furthermore, Homepage2vec concatenates all the individual features and processes them using fully connected layers. Thus, it is possible to obtain 100-dimensional embeddings by accessing the last hidden layer.

*MarkupLM* We used the MarkupLM (Li et al., 2022) base model^4^ and large model^5^ to extract the text and XPath from the HTML. We limited the number of nodes to 512, which is the model’s maximum processing capacity. We then translated each text node using the Libretranslate API^6^. We leveraged the MarkupLM model to embed each node and took the mean over all nodes of each HTML to obtain the embedding for the HTML. The final embedding has a dimension of 768. The MarkupLM model incorporates four types of embeddings: word, position, token, and XPath. To evaluate the impact of these different inputs, we compare the results of the two most influential configurations: one that combines word and position embeddings (MarkupLM-Word) and another that combines XPath and position embeddings (MarkupLM-XPath).

*Header Section Embedding* A website typically includes a header with the structure of a website including the main topics and subtopics of the website. Based on predefined rules we extracted this header. We then extracted the text and embedded it with a multilingual BERT-based sentence embedder. The embedding has 768 dimensions.

*Term Frequency - Inverse Document Frequency (TF-IDF)* To contextualize advanced neural approaches, we applied a conventional TF-IDF analysis to the textual content of the websites. To minimize the dimensionality of the vector representations, we translated the webpages into English and selected the 500 most informative words. We used this way of vector representation for both Support Vector Machine (SVM) classification as well as for a Feedforward Neural Network (FNN).

*SVM* We applied an SVM classifier to the webpage vector representations generated using TF-IDF. To obtain the optimal hyperparameters, we conducted a grid search on the training set.

*ResNet Embedding* As a simple visual embedding method we used the pre-trained ResNet18 model for embedding screenshots of the websites. We retrieved the screenshot of each website and embedded it with this ResNet18 model. This resulted in an embedding vector with 512 dimensions.

The TL approach involves using the models’ embeddings and training a FNN on top of the vector representation of the models. In the first hidden layer of the FNN, the embeddings are transformed into a 100-dimensional vector. The second hidden layer is the classification head. This architecture was also used for the classification in the original training of Homepage2vec, and we adopted the same activation functions and dropout rates.

### Scores and embedding evaluation

After the website contents are transformed into latent space by an encoder model, we can analyze how well the ground-truth labels (municipality vs. non-municipality) are naturally clustered. To evaluate the two clusters we used the Silhouette Score (Rousseeuw, 1987), the Davies-Bouldin Index (Davies and Bouldin, 1979) as well as an additional score we call Separation Distance General Score (SDG-score). In the TL case, we simply take the 100-dimensional vector from the hidden layer.

#### Silhouette score

The Silhouette Score first calculates the mean distance of one data point to all other data points in the same cluster (*a*(*i*) = average dissimilarity of i to all other objects of A). Then we take the average distance of the data point to each other cluster. The minimum average distance is selected to find the neighboring cluster. This is denoted as *b*(*i*) (= minimum average dissimilarity of i to all other objects of another cluster). If $a(i) < b(i)$, the silhouette score is $1 - a(i)/b(i)$ which is close to 1 when *a*(*i*) is a lot smaller than *b*(*i*) i.e. the data point is on average much closer to the points in its own cluster than to the neighboring cluster’s points. If $a(i) > b(i)$, then the Silhouette Score is $b(i)/a(i) - 1$ which is close to -1 if *a*(*i*) is a lot bigger than *b*(*i*) i.e. the data point is on average much closer to the points of the neighboring cluster. In the case of $a(i) = b(i)$, the Silhouette Score is set to 0. Thus the range of the silhouette score is between -1 and 1. The Silhouette Score is not robust to outliers (Rousseeuw, 1987).

We denote the *global* Silhouette Score as the average over all data points in the dataset and the *cluster* Silhouette Score as the average over all data points in each cluster.

#### Davies-bouldin index

The Davies-Bouldin index assesses clustering quality by comparing the compactness, defined as the average distance of each data point to its centroid within a cluster, with the separation between clusters. Compactness measures how closely data points within a cluster are grouped, while separation measures how distinct clusters are from each other. We calculate each possible cluster pair. We sum their respective compactness and divide by the separation, which is conventionally the distance between the centroids. Other distance metrics can be used. To get a cluster-specific score, we have the following formula:

1 $$\begin{aligned} R_i = max_{j\ne i}\frac{S_i + S_j}{d(C_i, C_j)} \end{aligned}$$

with $S_i$ and $S_j$ being the compactness score of cluster i and j and $d(C_i, C_j)$ being the distance between their centroids. We take the maximum value over each cluster pair (most challenging pairwise comparison) for the final Davies-Bouldin score for cluster i. The global Davies-Bouldin index is the average over all cluster-specific scores.

While we want to minimize the compactness score (numerator), we want to maximize the separation value (denominator). Thus, the score ranges from infinity to zero. Therefore, a smaller Davies-Bouldin index is desirable (Davies and Bouldin, 1979).

#### SDG score

The SDG score evaluates each cluster individually and takes the mean of every cluster value. It assesses whether each cluster is separable from the rest of the dataset by comparing the third quantile (*Q*3) of the within-cluster distance to its centroid with the first quantile (*Q*1) of outside cluster distances to the centroid. A high outside distance (high separation) and low within distance (compact cluster) results in a high score which is preferable. Thus, the SDG score ranges from 0 to infinity.

2 $$\begin{aligned} {S_{SDG}} = \frac{1}{C}\sum ^{C}_{k=1}{\frac{Q3(wcd(k))}{Q1(ocd(k))}} \end{aligned}$$

*C* represents the total amount of clusters. The function *wcd* returns a vector with length $J_{k}$ of distances of all observations within the *k*-th cluster to its centroid. The *ocd* function returns a vector of distances of observations outside the *k*-th cluster to the centroid of the $k-th$ cluster. The vector has the length $N-J_{k}$, with *N* being the number of all observations and $J_{k}$ the number of observations within the *k*-th cluster.

### Datasets

Some municipality websites were used in the MC-task dataset as well as in the PC-task data set. Otherwise, the used URLs are unique. The MCT dataset and the PCT dataset consist of municipality websites as well as websites that are similar to websites from municipalities. However, these similarities are of different kinds. The MCT dataset consists of municipality websites and websites closely related to each municipality. In most cases, the corresponding similar pages were tourist pages of the respective municipal websites. On the other hand, the similarity in the PCT dataset is the focus on organizations in the public sector that provide services to a customer. These consist of services that require the physical attendance of a customer or services that can be conducted completely online. Thus, the similarity means a similarity of service and information provision. Table 1 provides an overview on the datasets.

**Table 1:** Datasets used for the case studies

Task	Abbreviation	URLs	unique Domains	Classes
Municipality classification task	MCT	6396	1629	2
Public sector classification task	PCT	8065	4681	4
eService classification task	SCT	4085	372	2

#### MCT dataset

**Fig. 1:**
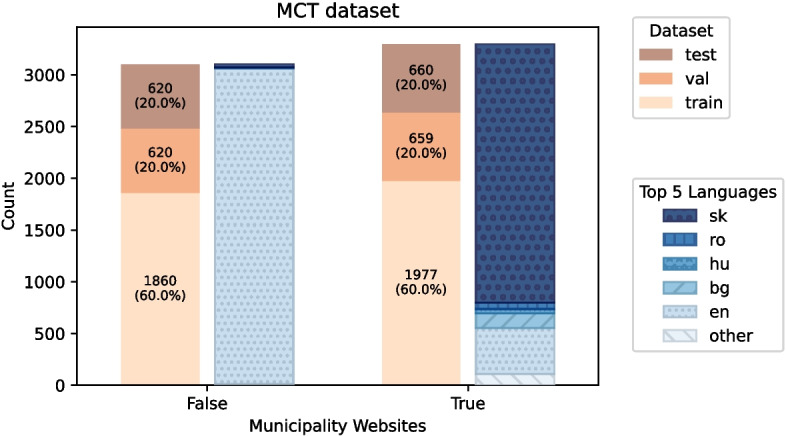
Distribution of MCT dataset showing classification, website language, and test, train and validation split sizes. True representing municipality websites and False representing non-municipality websites

Our dataset consists of 2901 municipality websites provided directly by the country administration and an additional 1349 municipality websites hand-labeled by domain exports. After dropping duplicates, the remaining municipalities are used to retrieve 3813 non-municipality websites using DuckDuckGo by querying <municipality name> + “tourism”. We use touristic websites since they share various characteristics with municipality websites and act as difficult negatives. The dataset contains websites of municipalities from ten different countries: Albania, Azerbaijan, Bulgaria, Croatia, Cyprus, Hungary, Romania, Serbia, Slovakia, and the United Kingdom. Only the landing page of each website was used for the embedding. After retrieving the HTML and embeddings of the Websites, the URLs without a result were dropped. This pruning results in a final dataset of 3100 municipality websites and 3296 non-municipality websites. Further details about the language distribution and the dataset splitting in the MCT dataset can be seen in Fig. 1.

#### PCT dataset

**Fig. 2:**
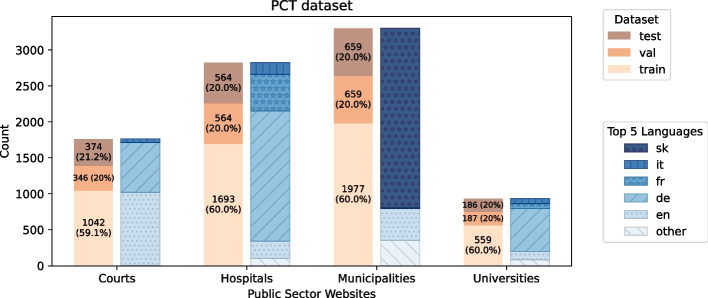
Distribution of PCT dataset showing classification, website language, and test, train and validation split sizes

The PCT dataset contains websites from courts, hospitals, universities, and municipalities. The data representing the class of municipalities is the same as in the MCT dataset. Thus the class consists of 3100 municipality websites. The URLs classified as courts, hospitals and universities are scraped data from Wikipedia tables and other documents that provide lists of URLs which can be assigned to these categories. The languages used are German, French, English, Spanish, and Italian. The different subgroups together result in a dataset of 10’031 URLs, of which 8’065 URLs were successfully embedded. Further details about the language distribution and the dataset splitting in the PCT dataset can be seen in Fig. 2.

#### SCT dataset

**Fig. 3:**
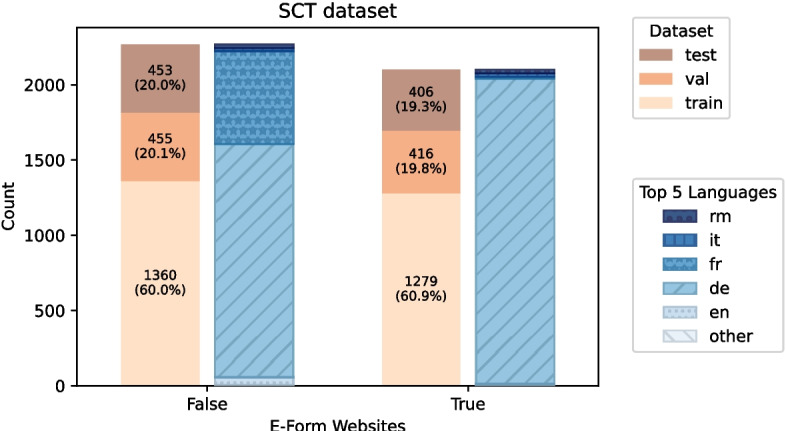
Distribution of SCT dataset divided in classification, website language, and dataset splitting

The dataset consists of a total of 5146 pages from 1129 different domains. The dataset consists of manually labeled URLs of Swiss municipality websites from the German, Italian, and French-speaking parts of Switzerland. The e-forms considered are either contact forms, subscriptions to newsletters and reminders, or online forms of services of a municipality, e.g. registering a pet animal. The reason we included contact forms as a form of service was that many municipalities use contact forms as a way of submission of certain service requests. Thus, the form provides a certain transaction of service between the user and the website owner, and is therefore included. However, we did not include any sophisticated search forms of a website since it does not lead to a transaction of service. The websites that could not be embedded were removed, resulting in the final dataset with 4085 websites. Further details on the SCT dataset can be seen in Fig. 3.

### Training, infrastructure and applicability

We generally used standard parameters from PyTorch for all the methods. We applied early stopping on the validation loss with a patience of 10 epochs for training. To ensure the robustness of the trained model, we implemented stratified K-Fold Cross-Validation with validation and test set. This involved dividing our dataset into 10 folds, which was split into a training, validation, and testing set with the proportions 60:20:20. Training and performance measurement was done on local and cloud computing environment. For local computing an 11^th^ Gen Intel(R) Core(TM) i9-11950H CPU with 16 cores, 32 GB RAM, and an NVIDIA RTX A2000 Laptop GPU with 4GB dedicated RAM was used. The cloud computing resources were Intel Core Processor (Broadwell, no TSX, IBRS) with 8 cores, 16 GB RAM, and a Tesla T4 GPU with 16GB dedicated RAM.

**Table 2:** Clustering scores for each embedding method and dataset showing the score without (P) and with transfer learning (TL) using an FNN on top of the frozen pre-trained models

		Silhouette		Davies-Bouldin		SDG	
Dataset	Embedding Method	P	TL	P	TL	P	TL
MCT	TF-IDF	0.100	0.790	3.090	0.282	1.103	5.336
	Header section	0.177	0.650	2.394	0.487	1.138	3.288
	ResNet	0.077	0.327	3.527	1.238	0.845	1.425
	Homepage2Vec	0.161	0.366	2.192	1.069	0.958	1.431
	H2V visual	0.299	0.461	1.347	0.854	1.235	1.802
	MarkupLM base*	0.175	0.765	2.213	0.320	1.012	4.273
	* Xpath	0.042	0.305	5.927	1.381	0.686	1.319
	* word	0.166	0.749	2.281	0.356	1.020	4.023
	MarkupLM large	0.179	0.771	2.158	0.316	1.081	4.238
PCT	TF-IDF	0.132	0.719	2.529	0.385	1.229	4.289
	Header section	0.121	0.448	3.082	0.885	1.017	1.984
	ResNet	-0.005	0.100	7.256	2.626	0.844	1.070
	Homepage2Vec	0.148	0.268	2.031	1.256	1.140	1.439
	H2V visual	0.294	0.393	1.278	0.962	1.620	1.944
	MarkupLM base*	0.101	0.678	3.164	0.468	0.906	3.556
	* Xpath	-0.011	0.033	11.325	4.467	0.645	0.802
	* word	0.101	0.669	3.219	0.493	0.912	3.491
	MarkupLM large	0.142	0.708	2.609	0.441	1.035	3.741
SCT	TF-IDF	0.031	0.565	5.916	0.596	0.941	2.169
	Header section	0.035	0.131	5.903	2.384	0.802	0.869
	ResNet	0.017	0.267	8.778	1.390	0.743	1.157
	Homepage2Vec	0.030	0.061	5.224	3.392	0.675	0.706
	H2V visual	0.020	0.034	5.460	4.801	0.628	0.642
	MarkupLM base*	0.017	0.498	7.105	0.672	0.739	1.919
	* Xpath	0.015	0.233	9.486	1.702	0.620	1.072
	* word	0.021	0.477	6.716	0.738	0.768	1.721
	MarkupLM large	0.021	0.504	6.722	0.684	0.774	1.921

The mean processing time per observation is also added. The best scores are underlined

## Case studies and results

We assess the different tasks separately and draw then a conclusion over all. In each subsection we discuss the general embedding evaluation scores on the zero-shot and transfer learning embeddings as well as the classification performance on each corresponding dataset.

### Results on the MCT dataset

The scoring of the embedding in Table 2 shows Homepage2vec with visual embedding to be the best embedding model when it comes to embedding domain-specific data without further adaptation. The combination of visual and textual embeddings seems to have an advantage. That is reasonable since certain distinctive features are only visually detectable by rendering images (e.g. municipalities tend to have a white background with an image of the municipality in the upper part of their websites). However, when only considering visual features, the model lacks the capability of distinctively building clusters. This is due to a lack of capability to understand the semantics of links and general text on the website. The light version of Homepage2vec without visual embedding performs worse than the other text-only-based approaches. The result of the ResNet embeddings, without further training, shows that it does not perform well in building clusters. The same is true for the pre-trained MarkupLM models and the header section embeddings. When using TL the TF-IDF with an added FNN layer performs best and has the biggest performance improvement. In both cases, the ResNet-based model is not able to compete with the other models. The high performance of Homepage2vec with visual embeddings does not translate to high performance with TL. This discrepancy may arise from the model’s original training, but crucially, the Homepage2vec embeddings are limited to 100 dimensions, while the embeddings used from other models are 500 or even more. A higher-dimensional embedding vector leads these models to have a higher performance jump from pre-trained to TL. An alternative approach with Homepage2vec could involve extracting embeddings from a different layer of the model. Additional embeddings of all models are shown in the appendix Fig. 9.

**Fig. 4:**
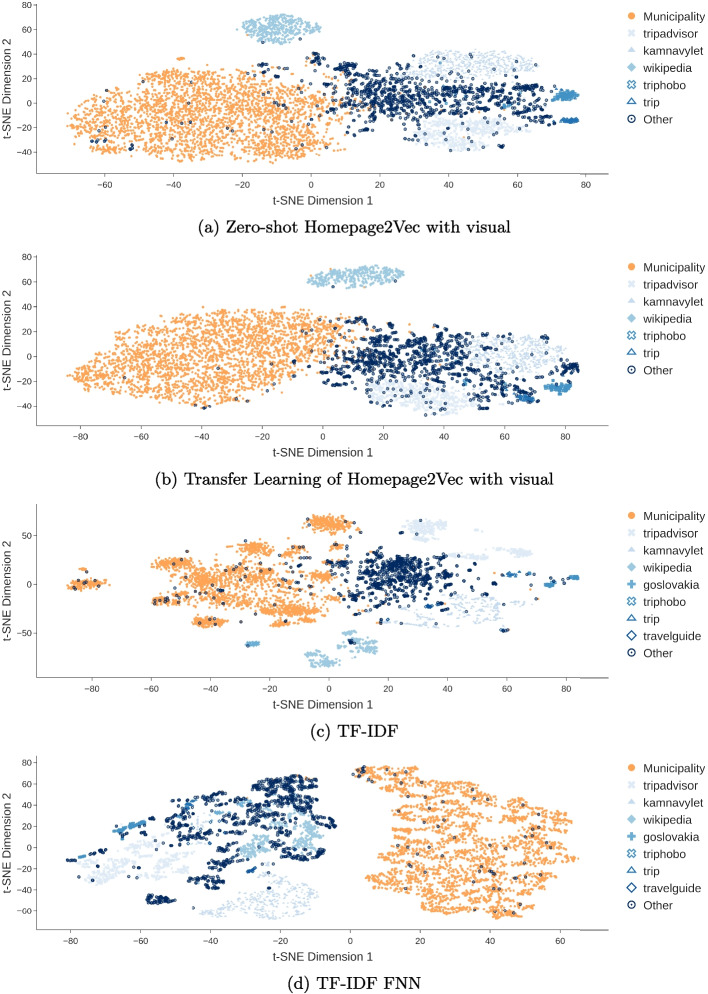
MCT dataset embeddings of municipality (orange / filled circle) and non-municipality websites (blues) labeled by most frequent domain

Figure 4 shows the separation of tourism websites from municipality websites. The embeddings with TL show less overlap of the clusters. The mean embedding time in seconds per URL is shown in Table 4. The time for the embedding includes fetching the HTML and screenshot, if needed, followed by the embedding method. In the case of MarkupLM and TF-IDF, the translation of the page content to English is also included in the time. The Header section embedding method performs best in terms of embedding time. The time correlates with the complexity of each model.

**Fig. 5:**
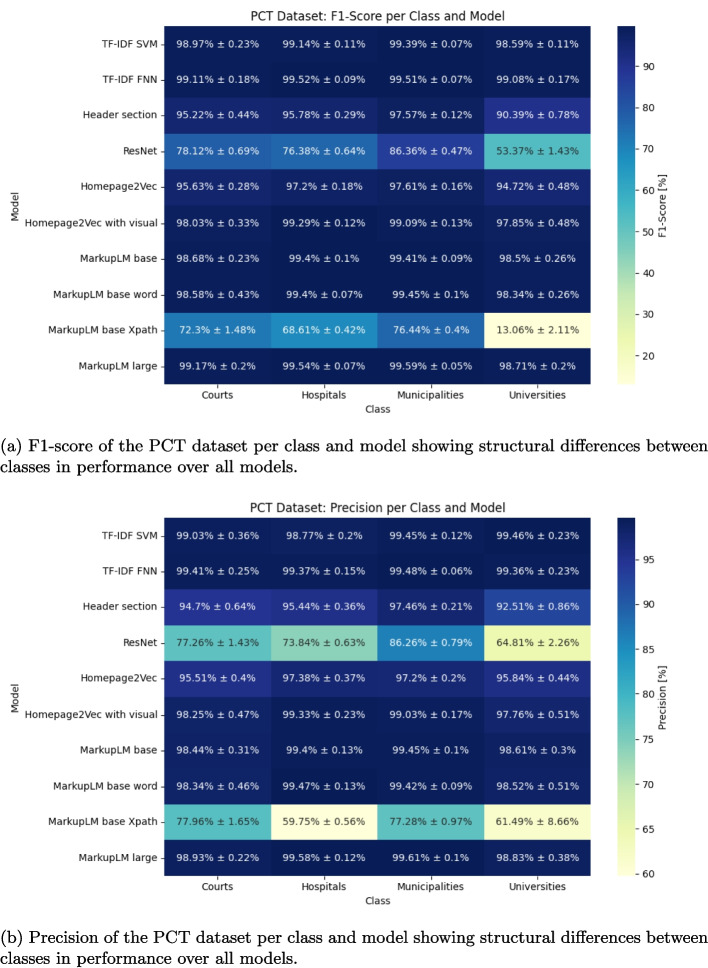
PCT multi-class classification performance

The TL results in discerning between municipality and non-municipality websites are shown in Table 4. We show the mean F1-Score and the precision alongside their respective margin of error. We prioritize precision in this context to minimize false positives. We use the standard error of the mean (SEM) to calculate the margin of error of the 10 K-Fold Cross-Validation. The TF-IDF-based models outperform all other approaches in both F1-score and precision. While this result may seem unexpected, it is not without explanation. Our findings indicate a general trend where text-based models surpass visually-oriented models. A detailed analysis of the impact of different MarkupLM inputs reveals that webpage text is a key factor in model performance. However, MarkupLM processes only a subset of a webpage, whereas TF-IDF captures the entire textual content and selects the most informative words, which is in our case 500 words. This gives TF-IDF a significant advantage, allowing even a simple FNN or SVM to outperform MarkupLM models, despite the latter being trained on a dataset of 24 million webpages (Fig. 5).

### Results on the PCT dataset

**Fig. 6:**
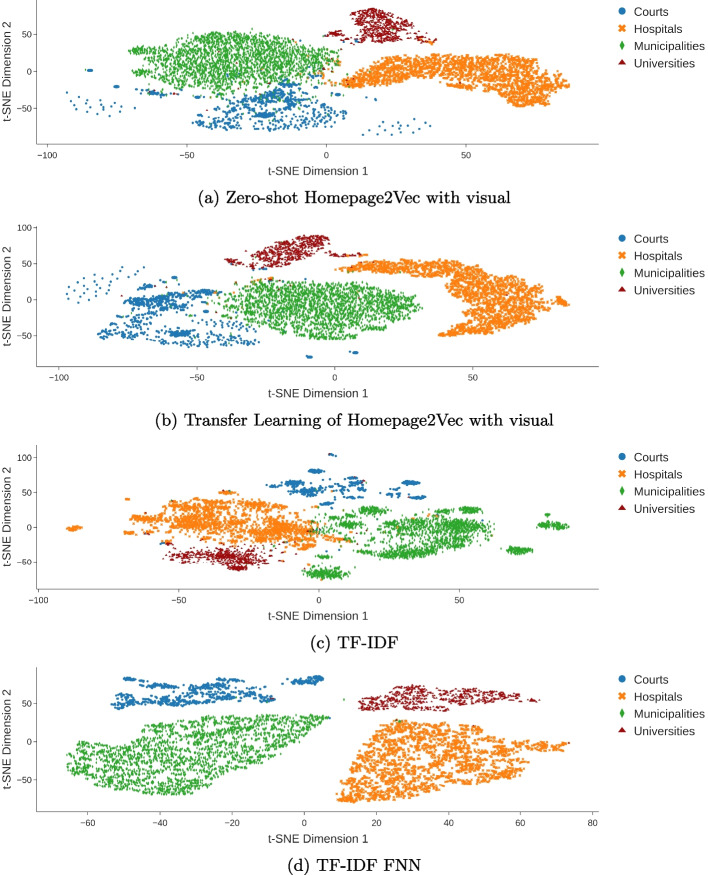
PCT dataset embeddings

Tables 2 and 4 show the scoring of the PCT dataset. We refrained from adding the time needed by each model to the table due to minimal changes to previously displayed results in Table 2. The results of binary classification, such as the MCT dataset, appear to be similar in multi-class classification. The mixed model performs best in the zero-shot approach again. As seen in in Fig. 6a the scatter plot shows a clear distinction. However, there are some court websites that are close and within the municipality cluster. This might be due to the fact that in different cases court websites are hosted by the corresponding municipality website. Thus, the website is a subpage of a municipality website and therefore visual structures might be very similar to municipality websites. Figure 6, which shows TF-IDF model vector representation, validates this thought. The court cluster is not as blurred in with the municipality cluster as in Fig. 6a. Thus the Homepage2Vec mixed model appears to give a reasonable representation of websites with a zero-shot approach.

In transfer learning scenarios, text-based approaches achieve the most significant improvements and overall best results. As shown in Table 2, TF-IDF consistently outperforms other models across all evaluation metrics. Similarly, in terms of classification performance, TF-IDF combined with an FNN achieves the highest scores, as presented in Table 4.

We observe a notable performance drop in the visual-based approach and the MarkupLM base XPath model when transitioning from binary classification of municipalities to multi-class classification. These models struggle to generalize across multiple classes compared to text-based approaches, highlighting the crucial role of textual components in HTML documents for classification.

Performance across different classes is relatively balanced across models. Excluding TF-IDF-based models, there appears to be a model-agnostic performance trend per class, as reflected in both F1-score per class (Fig. 5a) and precision per class (Fig. 5b). Municipality websites are the easiest to distinguish, while university websites present the greatest classification challenge. Additional embedding results for all models are provided in the appendix (Fig. 10).

**Fig. 7:**
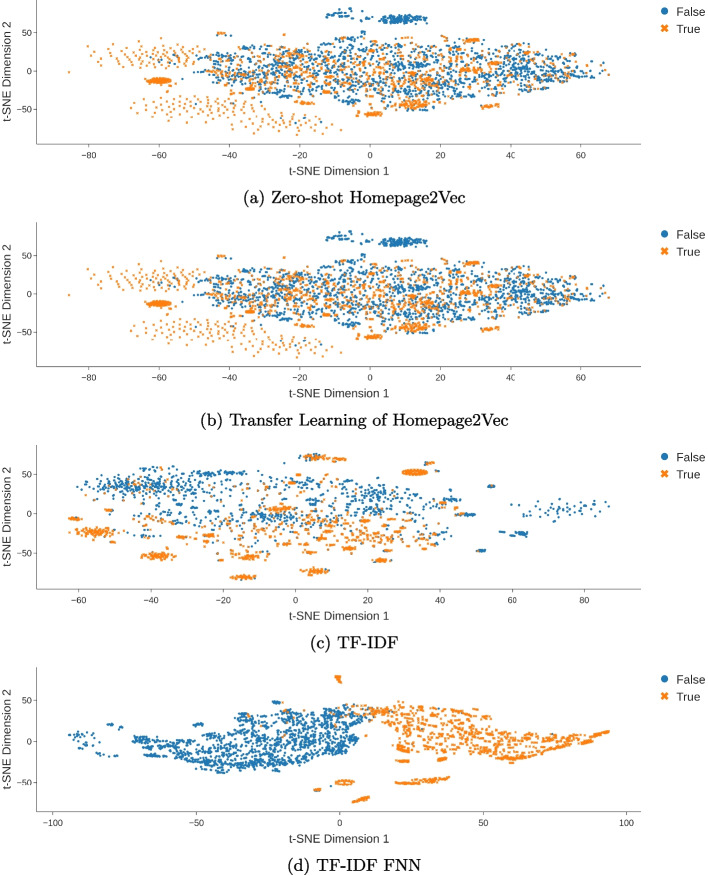
SCT dataset embeddings

**Fig. 8:**
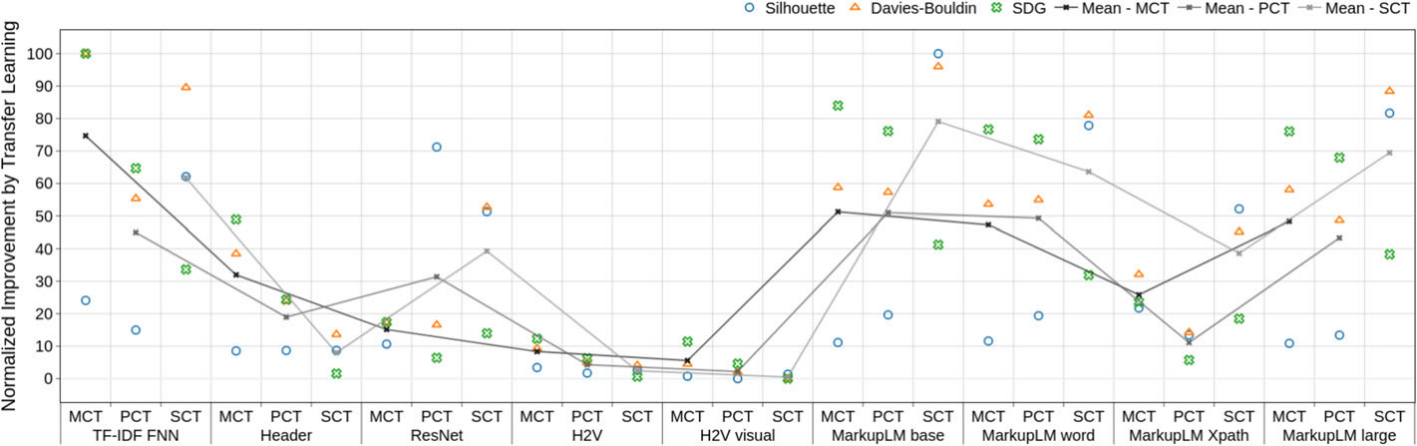
Improvement from Transfer Learning distinguished by data set and embedding method. Improvements are normalized by the scoring metric to a range of 0 to 100, resulting in 0 corresponding to the minimum improvement and 100 to the maximum improvement of one metric over all datasets and embedding methods. H2V stands for Homepage2Vec

### Results on the SCT dataset

Table 2 and 4 show the scoring of the SCT dataset. Classifying websites, whether they contain e-forms or not, appears to be mainly a visual task. However, when looking at the results we see that the text-only models outperform visual based only approach even in this task. When looking at the embedding scoring, the TF-IDF vector representaiton outperforms the others in most cases. When applying TL in general the TF-IDF vector representation achieves the best improvements. Table 4 shows that also the TF-IDF based models significantly outperform the other models in terms of classification scoring. The textual inputs appear to be the most important features for classification, eventhough not as crutial as in PCT. Unlike the MCT and PCT datasets, the visual model is placed third, which shows that the problem itself is also more of a visual nature than the other classification tasks.

When looking at the two most performant models concerning zero-shot and transfer learning (Fig. 7) we see that mixed model is not as adaptable to the new data as the textual models. Homepage2Vec appears to create two overlapping clusters with smaller cluster structures within. The model shows only a slight improvement when applying transfer learning and fails to create a single cluster per class. (Figure 7a, b). On the other hand, the TF-IDF based model appears to create multiple clusters with a tendency of True clusters rather being on one side and False clusters rather being on the opposite side. However, the model manages to create two distinguishable clusters when applying transfer learning (Fig. 7c, d). Based on Additional embeddings of all models are shown in the appendix Fig. 11.

### Model improvement and scalability

When comparing the performance improvement over the different datasets both MarkupLM models achieve the best improvement shown in Fig. 8. The Homepage2Vec models achieve the least improvement. There might be two reasons why this is the case. Firstly, the model needs much more data to substantially improve its performance. Secondly, the model is already at the limit of its capability to embed the complexity of the data. Although the MCT is a binary and the PCT is a multi-class classification problem there is a clear correlation between the mean performances over these datasets. This leads to the conclusion that the nature and complexity of these tasks are very similar. However, when looking at the SCT the improvement of the MarkupLM models are even bigger, but also the ResNet model has a bigger improvement. When comparing the performance with the classification results in Table 4, the ResNet model achieves a better result as well.

Comparing performance time across two different settings (as detailed in Section 3.3), all models - except Header Section and TF-IDF - benefit from increased dedicated RAM (Table 3). When considering only embedding time, TF-IDF achieves the shortest processing duration. The time analysis reveals that visual embeddings are the most computationally expensive. Consequently, the Homepage2Vec model significantly enhances processing efficiency when applied solely to text data.

Further reductions in processing time can be achieved by distributing dataset embedding across multiple cores, as demonstrated in the Header Section example. However, a weaker CPU leads to performance degradation.

Additionally, Table 3 indicates that the majority of processing time is spent on translation. This highlights the greater scalability of multilingual models, whereas TF-IDF models are more efficient when applied to websites in a single language or translation time is not considered (Table 4).

**Table 3:** Mean processing time per page for each embedding method comparing two different computing environments

	Local Computing (8GB VRAM)		Cloud Computing (16GB VRAM)	
Embedding Method	total time	excluded	total time	excluded
TF-IDF	7.5	0.1	17.6	0.1
Header section	3.1 / 0.5 $^{1}$	$-$	3.1 / 0.7 $^{1}$	$-$
ResNet	6.2	$-$	4.8	$-$
Homepage2Vec	0.4	$-$	0.2	$-$
H2V visual	9.0	$-$	5.1	
MarkupLM base	8.4	0.7	19.1	0.6
MarkupLM large	8.8	1.3	19.2	1.1

The time in seconds includes feature engineering and embedding. For the methods with translation, the total time and the time excluding the translation process (excluded) are noted. The time difference for each model measured across the dataset is negligible. The data shown is from the MCT dataset but is representative of the other datasets as well. The best scores are underlined

$^{1}$Header section without/with parallel processing using 8 cores

**Table 4:** Transfer learning (TL) scores of the models using K-Fold Cross-Validation with 10 k in percentages (± margin of error)

		True Class or Macro Average$^{1}$		Weighted Average	
Dataset	Embedding Method	F1-Score	Precision	F1-Score	Precision
MCT	TF-IDF & FNN	99.36 ± 0.10	99.46 ± 0.16		
	TF-IDF & SVM	99.27 ± 0.12	99.52 ± 0.18		
	Header section	98.10 ± 0.18	98.13 ± 0.27		
	ResNet	91.62 ± 0.40	91.67 ± 0.50		
	Homepage2vec	97.52 ± 0.19	97.17 ± 0.16		
	Homepage2vec visual	98.27 ± 0.21	98.33 ± 0.28		
	MarkupLM base	99.15 ± 0.12	99.21 ± 0.12		
	MarkupLM base xpath	90.42 ± 0.31	88.85 ± 0.47		
	MarkupLM base word	99.18 ± 0.05	99.30 ± 0.13		
	MarkupLM large	99.18 ± 0.09	99.36 ± 0.09		
PCT	TF-IDF & FNN	99.31 ± 0.09	99.41 ± 0.11	99.42 ± 0.07	99.42 ± 0.07
	TF-IDF & SVM	99.02 ± 0.11	99.18 ± 0.12	99.16 ± 0.09	99.16 ± 0.08
	Header section	94.74 ± 0.22	95.03 ± 0.25	95.82 ± 0.17	95.83 ± 0.17
	ResNet	73.56 ± 0.59	75.54 ± 0.65	78.02 ± 0.50	78.31 ± 0.52
	Homepage2Vec	96.29 ± 0.17	96.48 ± 0.16	96.88 ± 0.14	96.90 ± 0.14
	Homepage2Vec visual	98.57 ± 0.20	98.59 ± 0.20	98.88 ± 0.14	98.89 ± 0.14
	MarkupLM base	99.00 ± 0.12	98.98 ± 0.13	99.21 ± 0.09	99.21 ± 0.09
	MarkupLM base xpath	57.60 ± 0.84	69.12 ± 2.13	65.86 ± 0.51	69.41 ± 0.99
	MarkupLM base word	98.94 ± 0.16	98.94 ± 0.21	99.19 ± 0.11	99.20 ± 0.11
	MarkupLM large	99.25 ± 0.09	9.23 ± 0.12	99.42 ± 0.06	99.42 ± 0.06
SCT	TF-IDF & FNN	96.08 ± 0.24	95.76 ± 0.37		
	TF-IDF& SVM	96.30 ± 0.26	96.48 ± 0.26		
	Header section	82.51 ± 0.61	82.66 ± 0.79		
	ResNet	87.05 ± 0.63	88.82 ± 0.88		
	Homepage2Vec	84.58 ± 0.77	85.69 ± 0.99		
	Homepage2Vec visual	81.50 ± 0.59	83.12 ± 0.75		
	MarkupLM base	92.60 ± 0.50	93.35 ± 0.47		
	MarkupLM base xpath	83.97 ± 0.57	86.17 ± 0.62		
	MarkupLM base word	92.10 ± 0.41	93.50 ± 0.48		
	MarkupLM large	92.86 ± 0.48	93.16 ± 0.60		

The best scores are underlined

$^{1}$MCT and SCT Dataset: The F1-score and precision from the class True only. PCT Dataset: Macro average of the scores

## Conclusions

We compared different models on their capability of embedding HTML documents with high diversity and also tested their performance on classifying municipality websites in several classification tasks such as domain classifications as well as e-form detection on a website. We rated the embedding methods with several different clustering performance scores, which reward the capability of separating websites within a classification system. We compared the performance on the embedding of pre-trained models as well as the embedding performance of models with TL applied. We have seen that, based on the clustering scores, Homepage2Vec with a combined approach of using textual and visual features outperforms visual or text-only based models in most cases on a zero-shot approach. When applying TL and comparing the outputs of the last hidden layer as embeddings we have seen that TF-IDF based models had the biggest improvement and outperformed the mixed approach as well as the visual-based only. In general the text based TF-IDF models achieve the best results and outperform the other approaches on all datasets and classification tasks. Further analysis of the features used for training model trained on a markup language reveals that written text is a key factor. The ablation study of the MarkupLM model indicates that relying solely on text can sometimes compensate for a larger model that incorporates additional features. This phenomenon accounts for the strong performance of TF-IDF-based models. When comparing the models in a classification task on the MCT, PCT and SCT datasets TF-IDF based models outperforms the other approaches, with TF-IDF & FNN being the best most of the times. On the MCT dataset the model achieves a precision of 99.46 % and an F1-score of 99.36 %. On the PCT dataset the model achieves a weighted average precision of 99.42 % as well as a macro average precision of 99.41 % and a weighted average F1-score of 99.42 % as well as a weighted macro F1-score of 99.42 %. On the SCT dataset the model achieves a precision of 95.76 % and an F1-score of 96.08 %. When considering also process time we suggest the more simplistic Header section model, due to its multilinguality and scalability, or Homepage2Vec (text only) in the case of SCT. When processing data of only one language or if time does not play a role, TF-IDF outperform the other models in embedding, classification as well as process time.

## Future work

Often only a small percentage of page data indicates the affiliation of the page to its corresponding class e.g. form or input tag, which might be an indication for an e-form. The other part of the data may be considered as noise which potentially confuses a model or enlarges the amount of data needed to effectively train a model. Although, for example, the MarkupLM manages to filter this data to some extent, it uses a primitive approach to only consider a certain amount of tokens and discard the rest of the page data. Further work could be done in creating a segmentation rating mechanism that leads to reasonable and not random discrimination of data. Also depending on the classification task, certain pages might be affiliated to multiple classes. More insight in reasonable segmentation would also benefit a fine-grained classification task to assign each segment to only one class.

Additional research could also be done on how these different segments correlate with the embedding or classification of a website focusing on explainability. Text-based embeddings seem to be the best choice when it comes to TL for the classification of websites in a binary classification. We could enlarge the embeddings to not only focus on one site but rather on subsites of the domain as well. Many distinctive features of website categories are not immediately visible at the top level but become apparent at deeper levels of crawling. A comparison of models that also consider linked sites could be conducted.

To encourage the model to spread out the embeddings more effectively, one could apply triplet or contrastive learning approaches as seen in SimCSE (Gao et al., 2021). This could be coupled with more sophisticated methods to handle outlier edge cases. One approach could be to crawl potentially hard-to-classify web pages as part of a dataset augmentation strategy. When it comes to the training of classification models, labeled data is a valuable asset. Additional research could explore semi-supervised learning and active learning in this specific context. The foundation of an efficient application is a reasonable embedding of a given website which we have demonstrated is achievable.

## Figures and Tables

**Fig. 9:**
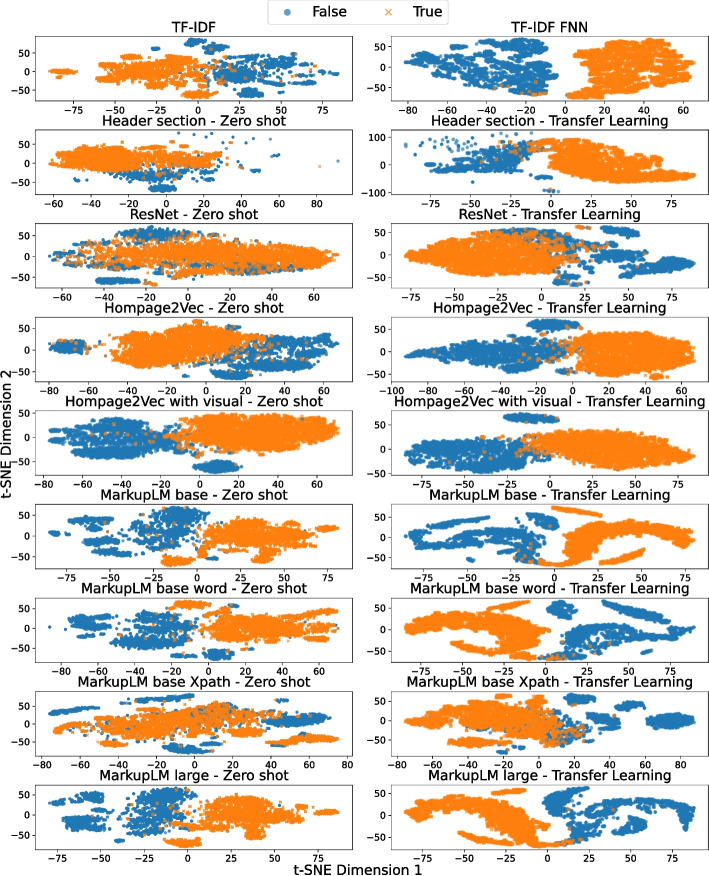
MCT Dataset: t-SNE Visualization of Embeddings. Two dimensional representation of the embeddings of each model with zero-shot embedding on the left side and transfer learning embedding on the right side

**Fig. 10:**
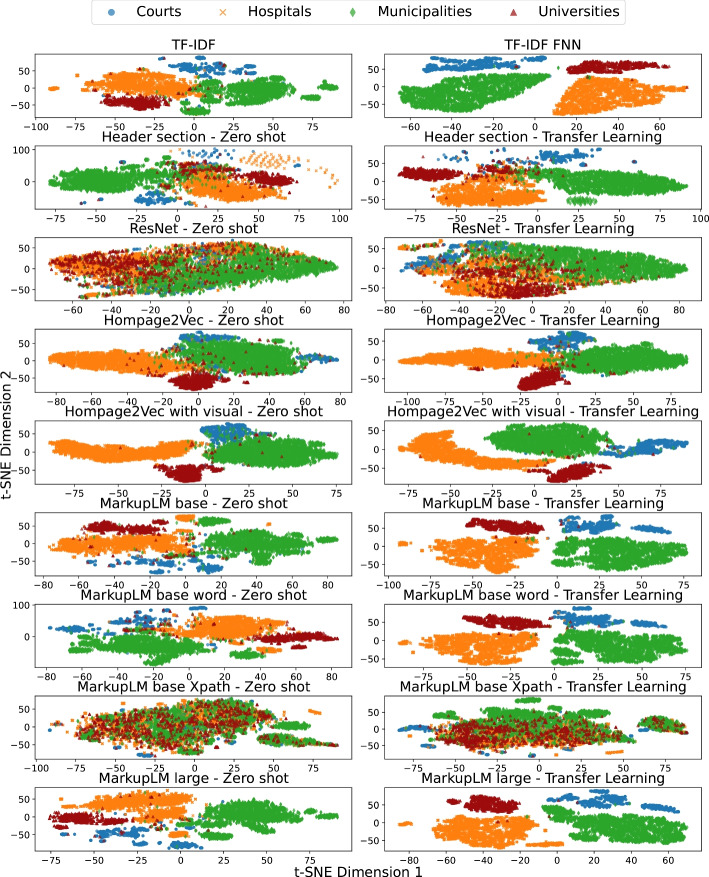
PCT Dataset: t-SNE Visualization of Embeddings. Two dimensional representation of the embeddings of each model with zero-shot embedding on the left side and transfer learning embedding on the right side

**Fig. 11:**
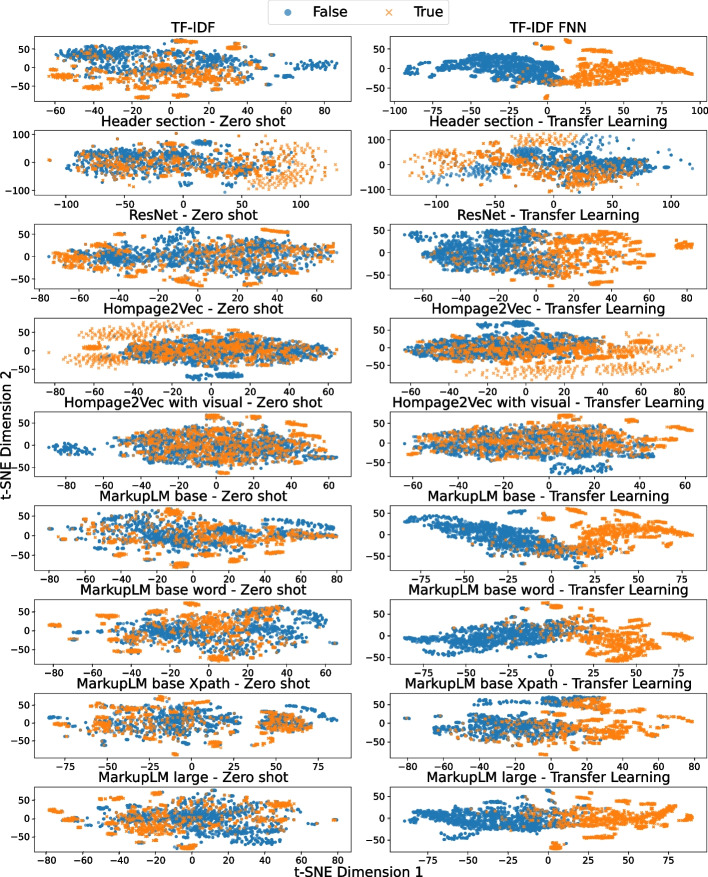
SCT Dataset: t-SNE Visualization of Embeddings. Two dimensional representation of the embeddings of each model with zero-shot embedding on the left side and transfer learning embedding on the right side

## Data Availability

The data will be published on the project homepage https://www.digilog-project.org/ but temporarely on https://drive.google.com/file/d/1Bn0FKHfTALJO0F_afHoW7IvcMmLOJCMa/view?usp=drive_link

## Footnotes

1 https://www.digilog-project.org/

2 https://github.com/JasminSaxer/website_embedding_benchmarks

3 https://pypi.org/project/homepage2vec/

4 https://huggingface.co/microsoft/markuplm-base

5 https://huggingface.co/microsoft/markuplm-large

6 https://libretranslate.com/
